# Transcriptome sequencing and expression analysis in peanut reveal the potential mechanism response to *Ralstonia solanacearum* infection

**DOI:** 10.1186/s12870-024-04877-0

**Published:** 2024-03-21

**Authors:** Xiao Wang, Feiyan Qi, Ziqi Sun, Hongfei Liu, Yue Wu, Xiaohui Wu, Jing Xu, Hua Liu, Li Qin, Zhenyu Wang, Suling Sang, Wenzhao Dong, Bingyan Huang, Zheng Zheng, Xinyou Zhang

**Affiliations:** 1 College of Agronomy, Shenyang Agricultural University, Shenyang, 110866 China; 2 The Shennong Laboratory, Institute of Crop Molecular Breeding, Henan Academy of Agricultural Sciences, National Innovation Centre for Bio-Breeding Industry, Key Laboratory of Oil Crops in Huang-Huai-Hai Plains, Ministry of Agriculture, Henan Provincial Key Laboratory for Oil Crops Improvement, Zhengzhou, 450002 China; 3 Henan Academy of Agricultural Sciences, Institute of Plant Protection, Zhengzhou, 450002 China

**Keywords:** Peanut, Bacterial wilt, Full-length transcriptome, RNA-sequencing, Plant-pathogen pathway, Glutathione metabolism, Candidate gene

## Abstract

**Background:**

Bacterial wilt caused by *Ralstonia solanacearum* severely affects peanut (*Arachis hypogaea* L.) yields. The breeding of resistant cultivars is an efficient means of controlling plant diseases. Therefore, identification of resistance genes effective against bacterial wilt is a matter of urgency. The lack of a reference genome for a resistant genotype severely hinders the process of identification of resistance genes in peanut. In addition, limited information is available on disease resistance-related pathways in peanut.

**Results:**

Full-length transcriptome data were used to generate wilt-resistant and -susceptible transcript pools. In total, 253,869 transcripts were retained to form a reference transcriptome for RNA-sequencing data analysis. Kyoto Encyclopedia of Genes and Genomes pathway enrichment analysis of differentially expressed genes revealed the plant-pathogen interaction pathway to be the main resistance-related pathway for peanut to prevent bacterial invasion and calcium plays an important role in this pathway. Glutathione metabolism was enriched in wilt-susceptible genotypes, which would promote glutathione synthesis in the early stages of pathogen invasion. Based on our previous quantitative trait locus (QTL) mapping results, the genes *arahy.V6I7WA* and *arahy.MXY2PU*, which encode nucleotide-binding site-leucine-rich repeat receptor proteins, were indicated to be associated with resistance to bacterial wilt.

**Conclusions:**

This study identified several pathways associated with resistance to bacterial wilt and identified candidate genes for bacterial wilt resistance in a major QTL region. These findings lay a foundation for investigation of the mechanism of resistance to bacterial wilt in peanut.

**Supplementary Information:**

The online version contains supplementary material available at 10.1186/s12870-024-04877-0.

## Background

Cultivated peanut (*Arachis hypogaea* L.) is an economically important oil crop. Peanut is widely grown in tropical and subtropical regions, where bacterial wilt (BW) is a serious disease that affects peanut yield and quality [1, 2]. *Ralstonia solanacearum* is a soil-borne gram-negative bacterium responsible for bacterial wilt disease in a broad range of host plants. Because biological and agricultural control methods are ineffective against BW, it is vital to breed a variety of cultivars with genetic resistance to BW. Therefore, identification of resistance genes and elucidation of the resistance mechanism is of utmost importance.

Several quantitative trait loci (QTL) that confer resistance to BW in peanut have been identified, of which a major QTL is located on chromosome B02 [3–6]. In addition, major QTLs located in the LG1 and LG10 linkage groups explain 12-21% of the phenotypic variation [7]. Most previous studies of BW in peanut have been based on QTL mapping, whereas few studies have explored the pathways involved in peanut resistance to BW. Chen et al. [8] conducted a RNA-sequencing (RNA-seq) analysis of susceptible (S) and resistant (R) peanut genotypes after inoculation with *R. solanacearum*, indicating that down-regulation of primary metabolism contributed to the difference in resistance between the R and S genotypes. The peanut cultivars ‘Yuanza 9102’ and ‘Xuzhou 68 − 4’ were subjected to RNA-seq analysis and the locus *arahy.5D95TJ* was identified as a candidate gene by quantitative real-time PCR (qRT-PCR) analysis [9]. The defense gene *AhDef2.2* was indicated to contribute to resistance to BW in peanut from a transcriptome analysis [10]. Currently, the BW resistance genes that have been identified in plant species comprise the *Arabidopsis thaliana* BW resistance gene *RRS1* [11] and *ERECTA* [12]; *CaLRR-RLK1* [13] and *CaLRR51* [14] are involved in the response to BW in pepper (*Capsicum annuum*); and overexpression of peanut *AhRLK1* [15] and *AhRRS5* [16] in tobacco (*Nicotiana tabacum*) enhances BW resistance. However, further research to identify resistance genes in peanut is needed.

In the course of their long-term co-evolution, plants have evolved a multi-layered defense system to resist infection by pathogenic microorganisms. Plant immune systems have been divided into two main categories: pattern-triggered immunity (PTI) and effector-triggered immunity (ETI) [17, 18]. The PTI system employs pattern recognition receptors on the cell surface to recognize the pathogen-associated molecular patterns (PAMPs). Successful pathogens release toxic proteins into plant cells that attack the host plant’s immune system to enable the pathogen to invade additional cells. Plants recognize pathogen effectors directly or indirectly by means of nucleotide-binding site-leucine-rich repeat receptor (NBS-LRR) proteins in cells [19–22], triggering the plant secondary immune system-ETI to activate a stronger immune response against pathogen infection [17, 18]. The PTI and ETI systems reinforce each other in plant immunity, which is of considerable importance for plant disease resistance and crop improvement [23, 24].

Meline et al. [25] performed a metaRNA-seq analysis and a series of experiments to validate that, in BW-resistant tomato (*Solanum lycopersicum*) genotypes, defense and growth are employed simultaneously to combat BW, whereas susceptible genotypes are infected by BW likely because of reduced tolerance of water stress. Chen et al. [26] reported that tomato BW resistance may be focused on plant-pathogen interaction pathways, plant hormone signal transduction pathways, and MAPK signaling pathways. With regard to tobacco, different studies using different materials and methods have identified the involvement of the same pathway in BW resistance, namely the phenylpropane pathway [27–29], but also different pathways, including glutathione metabolism [28] and hormone-related pathways [29]. Phytohormones play a critical role in eggplant (*Solanum melongena*) resistance to BW as indicated by transcriptome and metabolome analyses [30]. Dual RNA-seq has revealed that inhibition of ethylene synthesis, promotion of photosynthesis, up-regulation of polysaccharide metabolism, and strengthening of cell wall defense can prevent the invasion of *R. solanacearum* in pepper [31]. Arabidopsis and tomato respond to *R. solanacearum* infection by increasing the activity of pyruvate decarboxylases (PDCs). Thus, plant PDC-mediated metabolic pathways are enhanced to improve plant resistance to BW [32].

In this study, two peanut cultivars exhibiting either resistance or susceptibility to BW, together with their derivative recombinant inbred lines (RILs), were used for full-length transcriptome and RNA-seq analyses. The transcripts located in the target region of the genome were analyzed and two transcripts expressed in resistant genotypes may have a vital function in preventing pathogen invasion.

## Results

### Phenotype after inoculation with *R. solanacearum*

**Fig. 1:**
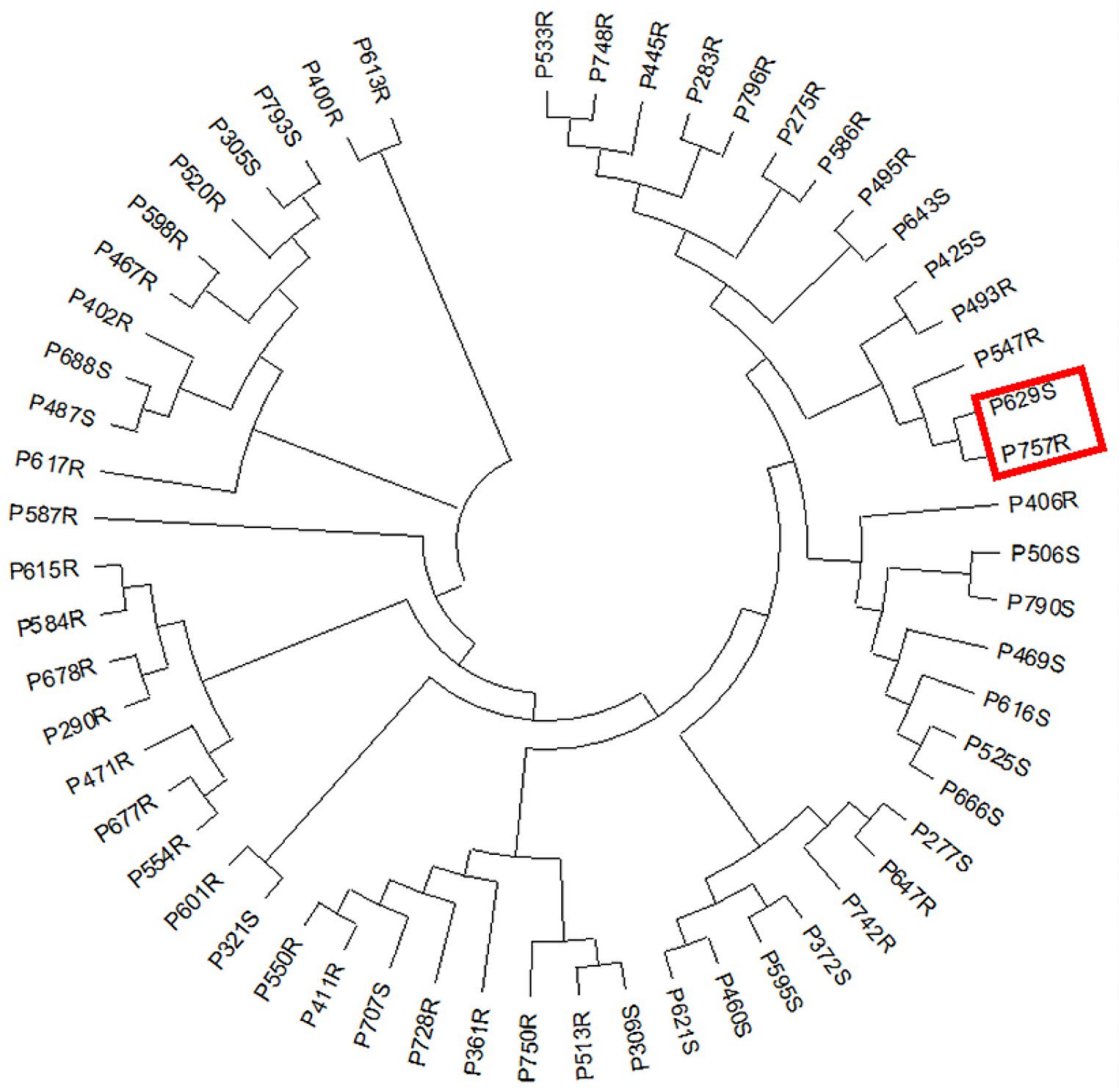
Phylogenetic tree for 57 peanut materials showing extreme resistance/susceptibility to bacterial wilt. Twenty-one resistant and 36 susceptible materials were clustered based on the dRAD sequencing data. The red rectangle indicates the materials chosen

**Fig. 2:**
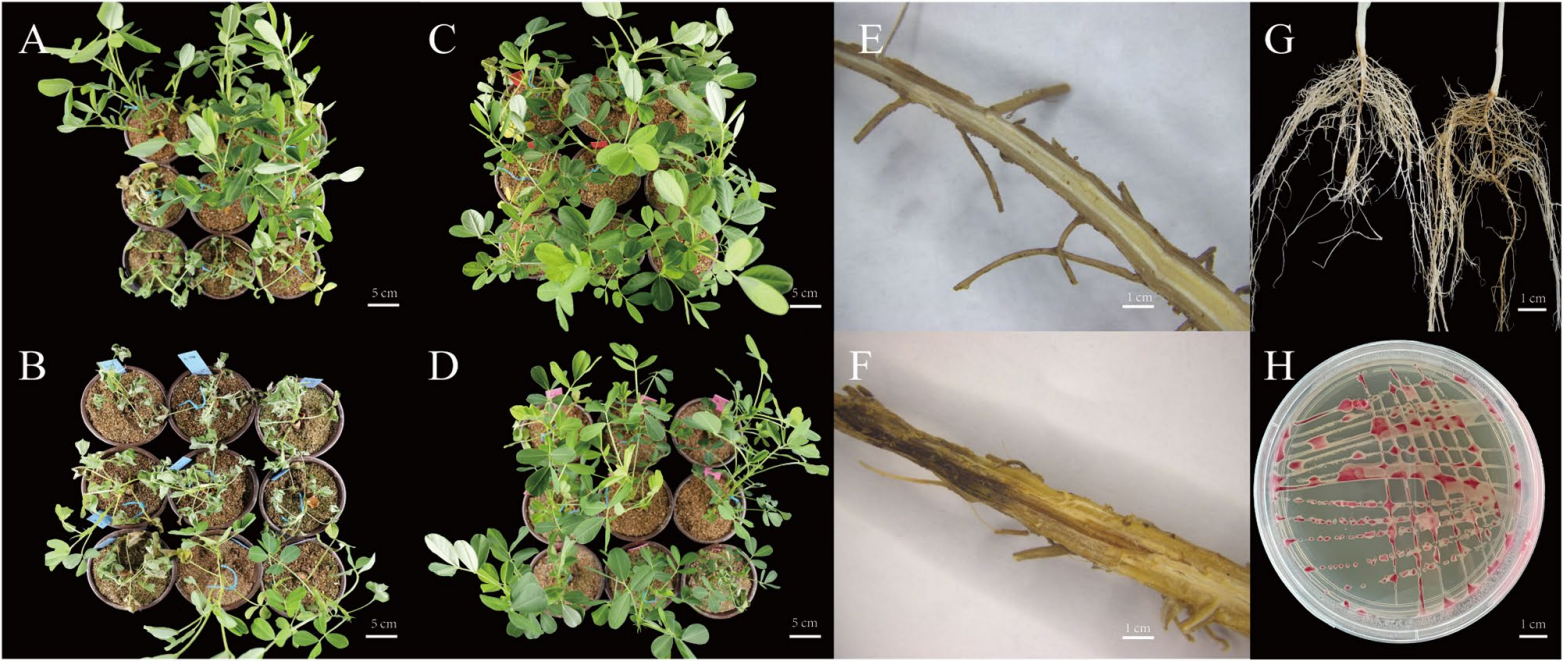
Peanut plant phenotype after inoculation with *Ralstonia solanacearum*. A-D Phenotype of indoor-grown plants of wt09-0023 (**A**), P629 (**B**), Yuanza 9102 (**C**), and P757 (**D**). Taproot of resistant (**E**) and susceptible (**F**) genotypes in longitudinal section. (**G**) Root phenotype of resistant (left) and susceptible (right) genotypes. (**H**) *Ralstonia solanacearum* cultured on TTC medium

Based on the digest restriction-site-associated DNA (dRAD) sequencing data, a cluster analysis was performed using 36 resistant and 21 susceptible strains of peanut, then the P757 (R) and P629 (S) lines were selected for further analysis (Fig. 1). The survival of seedlings of the four materials in the field under natural conditions [5] and indoors after inoculation with *R*. *solanacearum* was similar (Fig. 2; Table 1). At 15 days after inoculation with *R*. *solanacearum*, the peanut accession ‘wt09-0023’ (W) and S gradually showed leaf wilting (Fig. 2A,B), whereas the cultivar ‘Yuanza9102’ (Y) and R exhibited almost no symptoms (Fig. 2C,D). Compared with resistant plants, the roots of susceptible plants that were infected by bacteria were darker brown and the vascular tissue was blocked, thereby causing the plant to wilt (Fig. 2E-G).

**Table 1:** Percentage survival of seedlings of the four peanut materials grown in different environments

	Outdoor_2016	Outdoor_2017	Outdoor_2018	Indoor_2023
Yuanza9102(Y)	100	95.8	95	100
wt09-0023(W)	83.3	51.8	90	45
P757(R)	90	90	90	88.9
P629(S)	55.91	39.23	47.57	22.2

The *R. solanacearum* strain was isolated in Xinyang, Henan, and belongs to race 1 biovar 3. When cultured on triphenyl tetrazole chloride (TTC) medium, the *R. solanacearum* colonies were irregularly shaped, red in the center, and surrounded by white liquid (Fig. 2H).

### Full-length transcript analysis after incubation with *R. solanacearum*

According to the peanut botanical classification, the ‘Yuanza9102’ was divided into *Arachis hypogaea subspecies fastigiata*, while ‘wt09-0023’ belonged to *Arachis hypogaea subspecies hypogaea*, these two materials had 291,195 polymorphic SNPs [5]. In order to accurate analysis gene expression, we measured full-length transcripts analysis. We constructed two libraries for resistant samples (R1) and susceptible samples (S1), yielding 644,156 (73.49 Gb) and 1,547,052 (165.09 Gb) reads, respectively (Table S1). Quality control was performed on the original data to ensure the quality and reliability of the data analysis. The statistics for the subreads after quality control are shown in Table S2. In total, 596,303 and 1,411,238 circular consensus sequences (CCSs) were identified in the R1 and S1 samples, respectively, and classified as full-length based on the presence of 5′ primers, 3′ primers, and the poly(A) tail (Table S3). After clustering and removal of all redundant full-length non-chimeric (FLNC) sequences, and correction with the Arrow software, 53,927 (for R1) and 104,003 (for S1) polished consensus sequences were obtained. The consensus reads length ranged from 59 to 8817 and from 59 to 8563 for R1 and S1, respectively (Table S4).

**Fig. 3:**
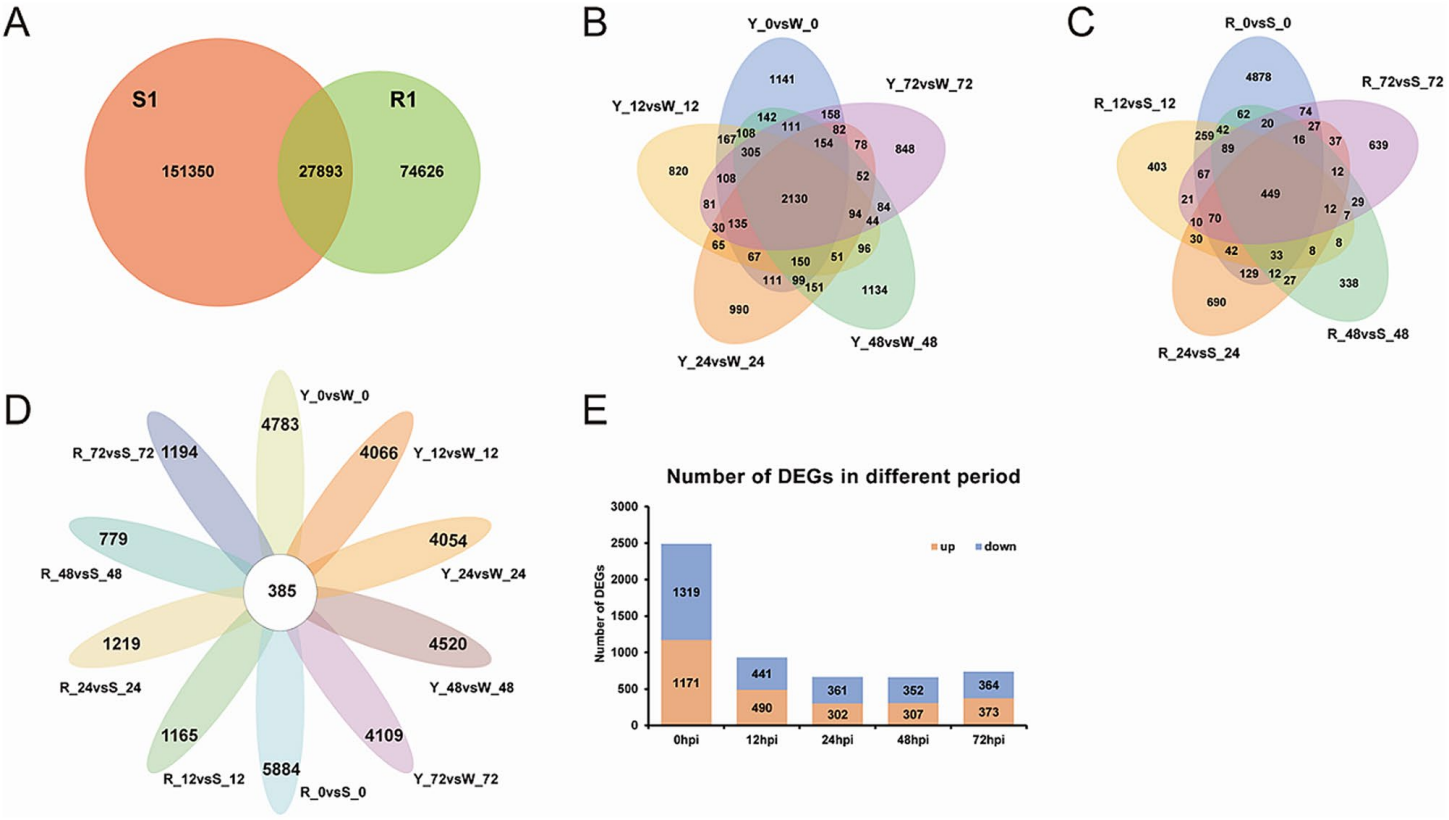
Comparative analysis of differentially expressed transcripts in different peanut materials. (**A**) Venn diagram of R1 and S1 transcripts. (**B**) Differentially expressed genes (DEGs) of Yuanza 9102 (Y) and wt09-0023 (W) in five periods after inoculation. (**C**) DEGs of P757 (R) and P629 (S) in five periods after inoculation. (**D**) DEGs for 10 comparisons. (**E**) Up-regulated and down-regulated DEGs in five periods after inoculation

The CD-HIT software was used to cluster the corrected transcripts that showed at least 95% similarity between the sequences to eliminate redundancy. Transcriptome cluster analysis was performed again by combining the R1 and S1 sequences after redundant sequences were removed. The unique and common transcripts among all samples were analyzed according to the clustering results and Venn diagrams were generated based on the transcriptome analysis results. S1 included 151,350 unique transcripts and R1 comprised 74,626 unique transcripts, and 27,893 transcripts were common to R1 and S1 (Fig. 3A).

### Gene function annotation of full-length transcript analysis

After removal of redundant sequences, 103,025 and 179,882 transcripts were retained for R1 and S1 with mean lengths of 2751 bp (N_50_ = 3014, N_90_ = 1788) and 2650 bp (N_50_ = 2899, N_90_ = 1740) respectively. The transcripts were annotated by alignment with sequences in seven public databases, namely, the non-redundant protein sequence(nr) and nucleotide sequences(nt) from NCBI, Pfam, KOG(eukaryotic Ortholog Groups), Swiss-prot, Kyoto Encyclopedia of Genes and Genomes (KEGG), and Gene Ontology (GO) databases. Among the transcripts, 99.77% and 99.66% were annotated by at least one database for R1 and S1, respectively. In addition, 42.83% and 41.35% of the total number were annotated by all databases for R1 and S1, respectively (Fig. S1A,B).

### Full-length transcript structure analysis

Prediction of the protein-coding region in full-length transcriptome sequencing data is conducive to preliminary analysis of the genes. The distribution of CDS lengths in R1 and S1 is shown in Fig. S1C,D. The annotation of the 30 TF families with the largest number of transcripts in each of R1 and S1 is shown in Fig. S1E,F.

### RNA-seq analysis of Y, W, R and S infected with *R. solanacearum*

To find out which genes have changed their expression level after being infected by *R. solanacearum* we conducted RNA-seq sequencing. The taproots of the four materials (Y, W, R and S) were collected at 0, 12, 24, 48, and 72 hpi; each sample comprised three replicates for RNA-seq analysis. In total, 60 RNAs were sequenced, yielding 400.39 Gb of raw data. After filtering, checking for sequencing errors, and assessing the GC content distribution, 390.64 Gb of clean data were retained. On average, the clean data had Q20 and Q30 scores of 97% and 93%, respectively, and a GC content of 44%, which demonstrated the high quality of the sequencing data (Table S5). Based on the fragments per kilobase of transcript per million reads (FPKM), principal component analysis and Pearson correlation analysis were used to assess the pattern of clustering of the biological replicates and samples. Compared with the data at 0 h post-incubation (hpi), the transcriptome changed markedly in response to inoculation with *R. solanacearum*, and almost all biological replicates clustered together, indicating the data showed a high degree of reliability (Fig. S2A,B).

### Differentially expressed gene identification and cluster analysis after the *R. solanacearum* infection

To explore the mechanism of peanut resistance to BW, differentially expressed genes (DEGs) were analyzed between Y and W, and between R and S for five periods (0, 12, 24, 48, and 72 hpi) under the criteria |log_2_ (fold change)| > 1 and *p*_adj_ < 0.05. With regard to Y vs. W, 5168, 4451, 4439, 4905, and 4494 DEGs were identified at 0, 12, 24, 48, and 72 hpi, respectively (Fig. 3B). The maximum number of DEGs between R and S was 6269 at 0 hpi, followed by 1604, 1579, 1550, and 1164 at 24, 72, 12, and 48 hpi, respectively (Fig. 3C). Interestingly, 385 DEGs were identified as common to all comparisons (Fig. 3D). All comparison groups were taken as differential gene sets after the union, and the 32,784 DEGs were clustered and a heatmap was generated (Fig. S2C).

To exclude the genetic background noise, the intersection of DEGs between the two parents and between the two progenies was considered for each period. The highest number of DEGs were identified at 0 hpi. In comparisons of the susceptible W and S with the resistant Y and R, 1319 transcripts were up-regulated and 1171 were down-regulated (Fig. 3E). The identified DEGs were subjected to further analysis.

### GO and KEGG enrichment analysis of DEGs

**Fig. 4:**
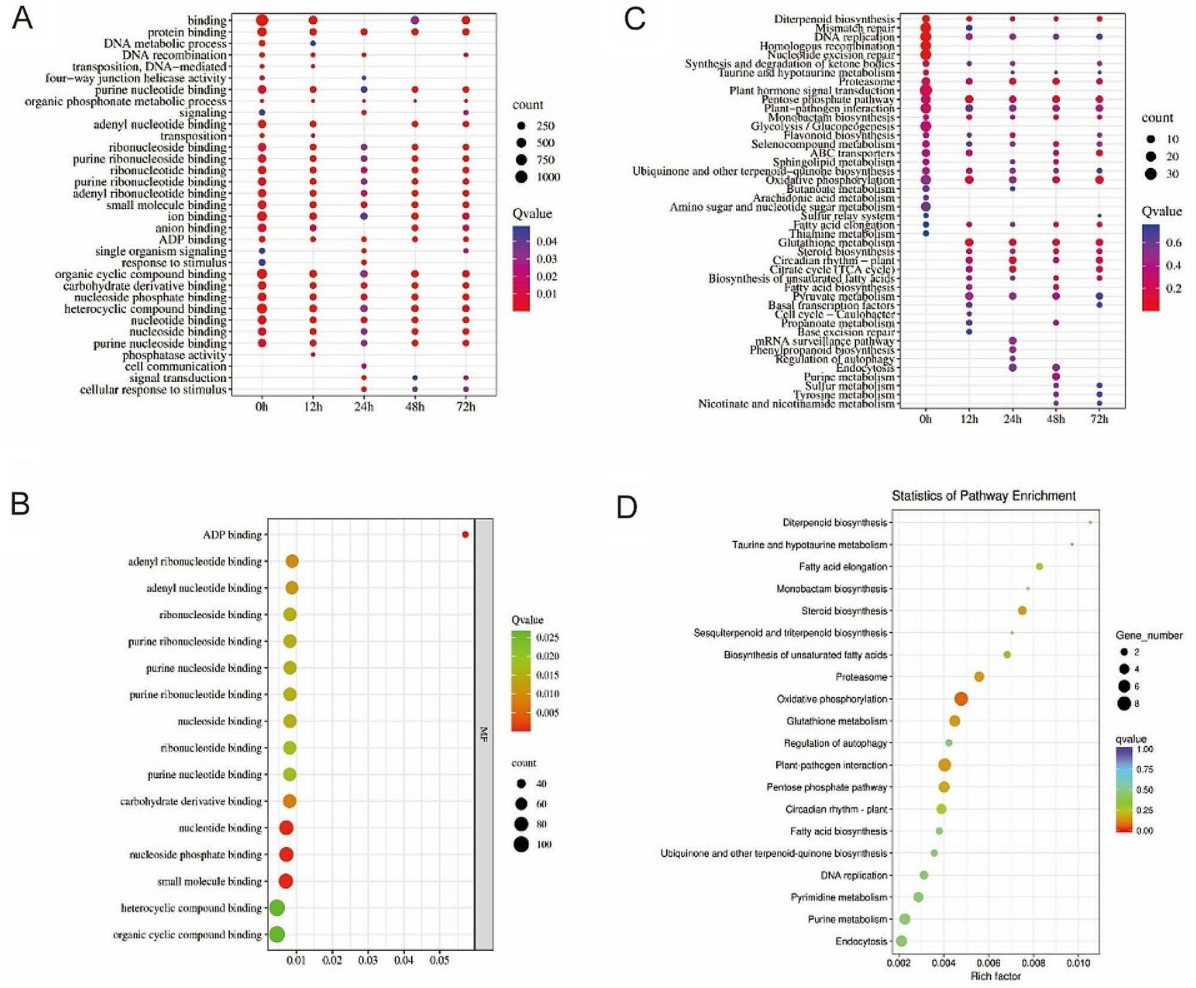
GO and KEGG enrichment analyses. (**A**) and (**C**) GO enrichment analysis. (**B**) and (**D**) KEGG enrichment analysis. (**A**) GO and (**B**) KEGG dotplots in five periods after inoculation. The color scale indicates significance (*Q*-value < 0.05). (**C**) Top 16 GO terms and (**D**) top 20 KEGG pathways for 10 groups of crossed DEGs

To understand the biological processes and molecular functions that changed after inoculation with *R. solanacearum*, a gene ontology (GO) analysis was performed using the intersected DEGs at the five periods for the four materials (Fig. 4A) and 385 DEGs in all comparisons (Fig. 4B). In the biological process category, cellular response to stimulus (GO:0051716), signal transduction (GO:0007165), single organism signaling (GO:0044700), signaling (GO:0023052), and cell communication (GO:0007154) were significantly enriched after inoculation, indicating that the plants resisted bacterial invasion by enhancing signal transduction between cells (Fig. 4A). In the molecular process category, purine nucleoside binding (GO:0001883), purine ribonucleoside binding (GO:0032550), purine ribonucleotide binding (GO:0032555), and purine nucleotide binding (GO:0017076), for example, were significantly enriched in all periods (Fig. 4A,B).

With regard to KEGG pathway enrichment analysis, diterpenoid biosynthesis (ko00904), DNA replication (ko03030), proteasome (ko03050), pentose phosphate pathway (ko00030), plant-pathogen interaction (ko04626), selenocompound metabolism (ko00450), ubiquinone and other terpenoid-quinone biosynthesis (ko00130), oxidative phosphorylation (ko00190), and fatty acid elongation (ko00062) were enriched in all periods (Fig. 4C,D). Glutathione metabolism (ko00480), steroid biosynthesis (ko00100), pyruvate metabolism (ko00620), and circadian rhythm-plant (ko04712) were enriched in all periods except at 0 hpi (Fig. 4C).

### Candidate gene analysis in a major QTL region for resistance to bacterial wilt

**Fig. 5:**
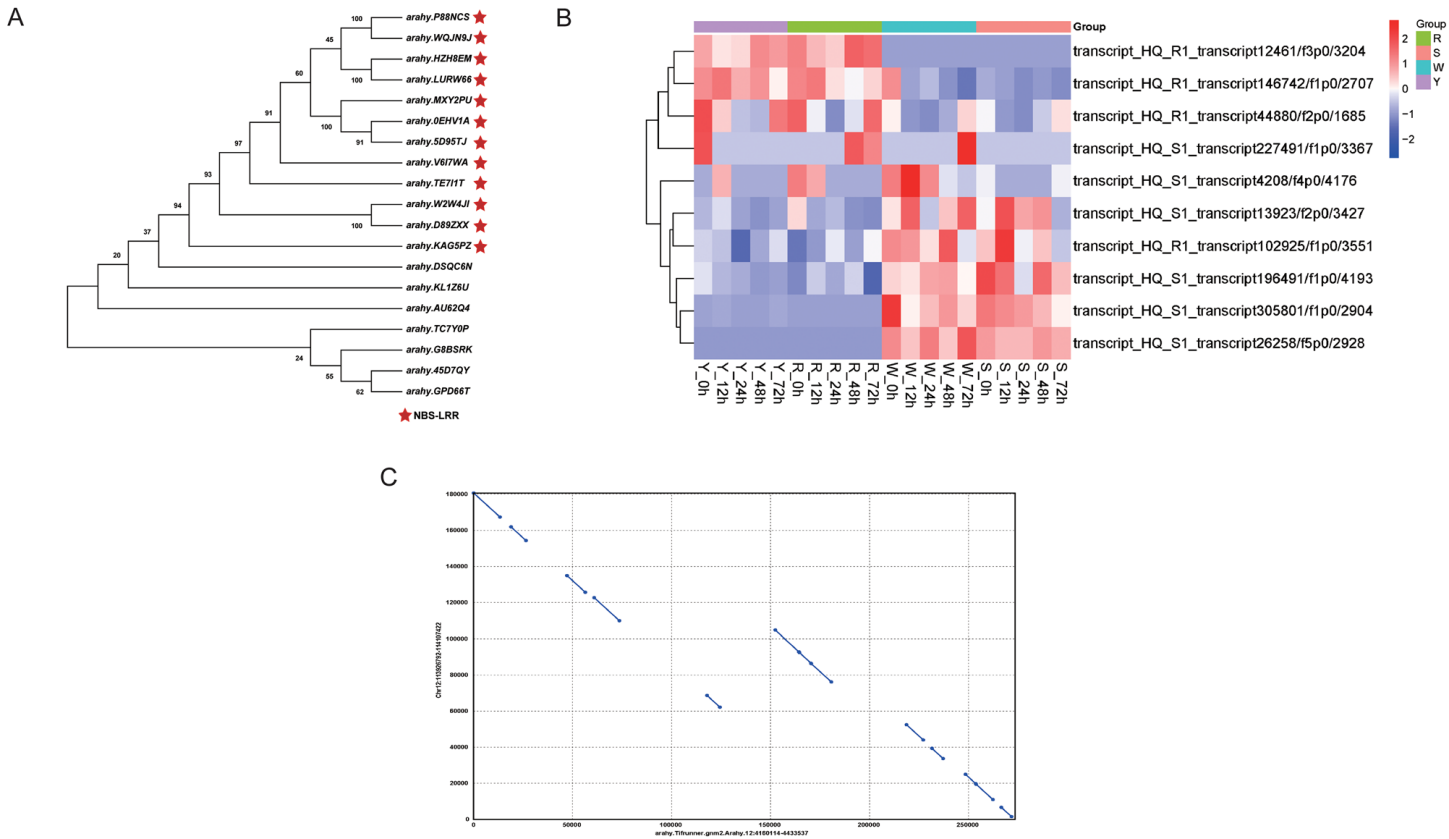
Candidate gene analysis in the target major quantitative trait locus (QTL) region for resistance to bacterial wilt. (**A**) Phylogenetic tree of candidate genes in the peanut Tifrunner reference genome. Red five-pointed stars indicate nucleotide-binding site-leucine-rich repeat receptor (NBS-LRR) genes. (**B**) Heatmap of R1 and S1 transcripts in the QTL region. (**C**) Collinearity analysis of the Tifrunner genome sequences and Yuanza9102 genome sequences

Our previous research showed that a major QTL for BW resistance was located in a 216.7 kb region on the LG12 based on a RIL population constructed from a cross between Y and W. This interval contained 19 genes of which 12 were annotated as NBS-LRR genes (Fig. 5A). However, further analysis of these genes revealed that several of them showed high sequence similarity. After removal of the highly similar duplicates, 15 genes were used for subsequent analysis. In the full-length transcriptome sequencing data for R1 and S1, six of the 15 genes were not expressed in both R1 and S1, four genes were expressed in both R1 and S1, and five genes were only expressed in S1 (Table 2). Among the R1 and S1 transcripts, the loci *arahy.WQJN9J* and *arahy.MXY2PU* corresponded to transcripts that showed low sequence similarity. A heatmap for the transcripts of the genes in the QTL region is shown in Fig. 5B. The reference genome sequence collinearity for the genes in the QTL region was analyzed (Fig. 5C). The *x*-axis 100,000-200,000 region corresponds to the *y*-axis 60,000-100,000 region; thus, the collinearity for this interval was poor. The genes *arahy.V6I7WA* and *arahy.MXY2PU* may be associated with BW resistance. The genes *arahy.MXY2PU* and *arahy.5D95TJ* showed high similarity (Fig. 5A).

**Table 2:** Transcripts corresponding to a reference genome gene ID

Number	Reference ID	R1 transcripts	S1 transcripts	Identity
1	*arahy.AU62Q4*	transcript_HQ_R1_transcript44880/f2p0/1685	transcript_HQ_S1_transcript82515/f4p0/1683	99.7%
2	*arahy.V6I7WA*	transcript_HQ_R1_transcript146742/f1p0/2707	transcript_HQ_S1_transcript305801/f1p0/2904	90.55%
3	*arahy.TE7I1T*		transcript_HQ_S1_transcript251764/f1p0/1404	
4	*arahy.0EHV1A*		transcript_HQ_S1_transcript196491/f1p0/4193	
5	*arahy.WQJN9J*	transcript_HQ_R1_transcript102925/f1p0/3551	transcript_HQ_S1_transcript13923/f2p0/3427	36.86%
6	*arahy.LURW66*		transcript_HQ_S1_transcript26258/f5p0/2928	
7	*arahy.MXY2PU*	transcript_HQ_R1_transcript12461/f3p0/3204	transcript_HQ_S1_transcript4208/f4p0/4176	59.2%
8	*arahy.G8BSRK*		transcript_HQ_S1_transcript82599/f3p0/1680	
9	*arahy.GPD66T*		transcript_HQ_S1_transcript227491/f1p0/3367	

### Plant-pathogen interaction pathways

**Fig. 6:**
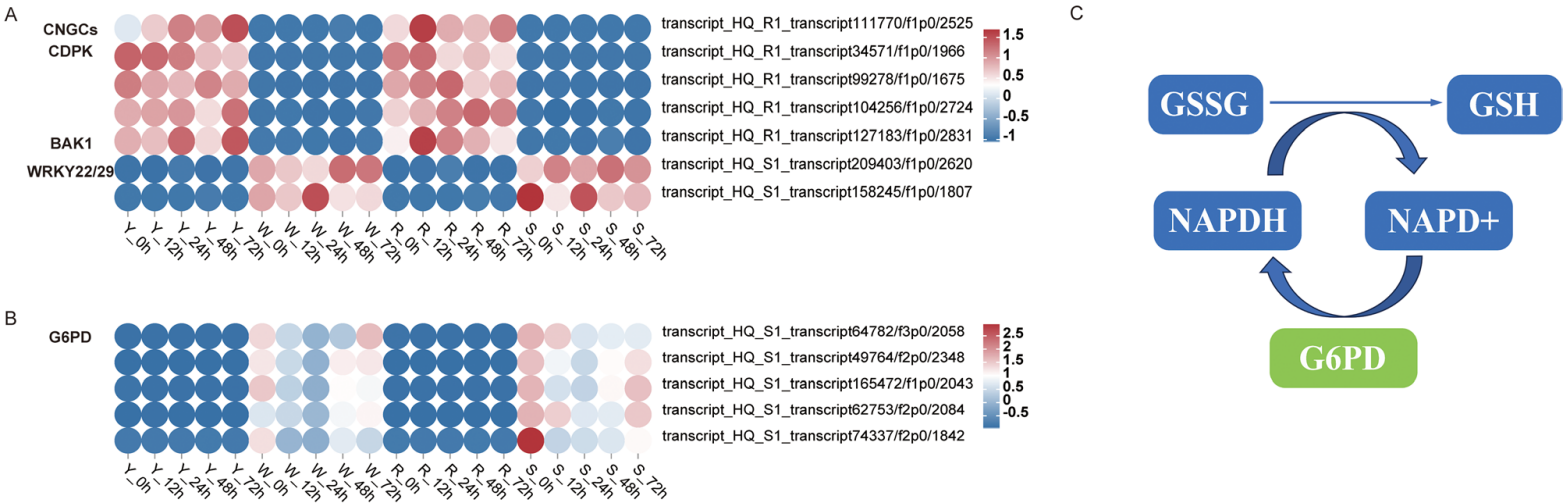
Heatmaps showing the relative expression of differentially expressed genes in different pathways. The color scale from red to blue indicates the relative expression level ranging from high to low. (**A**) Plant-pathogen interaction pathway, (**B**) glutathione metabolism pathway. (**C**) Schematic diagram of glutathione metabolism

Plant-pathogen interaction plays a vital role in preventing invasion of plants by pathogens. The PTI and ETI processes are included in this pathway, which reinforce each other to eliminate the pathogen [17]. In this pathway, several DEGs that play a critical role in plant immunity were identified. The expression levels of the transcripts of genes associated with plant-pathogen interaction are shown in Fig. 6A. The transcript_HQ_R1_transcript111770/f1p0/2525 (CNGCs) is required for Ca^2+^ signal transduction to promote opening of the cyclic nucleotide-gated ion channel on the plasma membrane [33]. A large family of calcium-binding regulatory protein kinases transcript_HQ_R1_transcript34571/f1p0/1966, transcript_HQ_R1_transcript99278/f1p0/1675, transcript_HQ_R1_transcript104256/f1p0/2724, and transcript_HQ_R1_transcript127183/f1p0/2831 (CDPK) participate in numerous aspects of plant growth and development, including Ca^2+^ signal transduction [34, 35]. An Arabidopsis LRR receptor-like protein kinase BAK1 (transcript_HQ_S1_transcript209403/f1p0/2620) negatively modulates brassinosteroid signaling [36]. Members of the WRKY gene family (transcript_HQ_S1_transcript158245/f1p0/1807) may play a negative role in pathogen defense [37, 38].

### Glutathione metabolism

Glutathione (GSH) plays a crucial role in protecting cells from oxidation and stress, primarily acting through the metabolic and signal transduction pathways to exert its physiological functions [39]. Glutathione is mainly involved in plant disease resistance, cell proliferation, root development, salt tolerance, protection against cold damage, and metabolic detoxification of a series of heterogenic elements (e.g., herbicides, sulfur dioxide, and ozone) and heavy metals [40]. Interestingly, in the present study, GSH metabolism was only identified in response to bacterial invasion of the susceptible genotypes (Fig. 6B,C). The transcripts function as glucose 6-phosphate dehydrogenase, which catalyzes NAPDH synthesis, and thus facilitates the generation of GSH [41–43]. These results indicated that the glutathione metabolism pathway may play an important role in the susceptibility of peanut genotypes to infection by *R. solanacearum* and wilting at an early stage of invasion.

### Validation of RNA-seq data by qRT-PCR analysis

To validate the reliability of the RNA-seq data, six transcripts that were differentially expressed in the four materials in the five periods were randomly selected for qRT-PCR analysis with three biological replicates. The results obtained from the qRT-PCR and RNA-seq analyses showed the same trends in different materials and periods, which demonstrated that the RNA-seq data were reliable (Fig. S3).

## Discussion

### Why not refer to the reference genome?

The reference genomes of *Arachis hypogaea* Tifrunner [44, 45] and Shitouqi [46] were published within the last 10 years, but both cultivars are susceptible to *R. solanacearum*. Because of the lack of reference genomic information for a resistant genotype, full-length transcriptome sequencing was performed to construct separate resistant and susceptible transcript pools. When referring to the Tifrunner or Shitouqi reference genomes, the resistance-associated transcripts located in the target major QTL region were filtered. The Tifrunner genome contains many identical genes in the QTL region and has low collinearity with the Yuanza 9102 genome (Fig. 5A, C), and thus the Tifrunner genome is unsuitable as a reference for further research. Therefore, we constructed a combination of the two pools of transcripts for subsequent analysis. Because of a lack of annotations for Yuanza 9102 reads, transcripts from the two pools cannot be corresponding as one gene like Table 2, only genes within the QTL interval were analyzed manually. Thus. no-reference analysis was applied in this study.

### The role of calcium in the plant immune system

As a second messenger, calcium plays an important role in activation of the plant immune system. Plants activate PTI by recognizing characteristic molecular patterns (PAMPs) from pathogenic microorganisms, and activate ETI by recognizing effector proteins of pathogenic organisms. The rapid, instantaneous intracellular flow of calcium ions induced by PAMPs is essential for full activation of PTI and ETI [47]. A point mutation in a unique calcium channel gene, *CNGC20*, leads to increase in the intracellular calcium concentration, which enhances the plant immune response and disease resistance [48]. WeiTsing, a calcium-permeable cation-selective channel, is transcriptionally activated in the pericycle in response to *Plasmodiophora brassicae* infection to prevent pathogen colonization in the stele [49]. Rice (*Oryza sativa*) immunity is suppressed by boosting reactive oxygen species scavenging through a Ca^2+^-sensor that is encoded by the *resistance of rice to disease 1* (*ROD1*) gene [50]. Thus, Ca^2+^-mediated pathways are closely associated with the plant immune system. In the present study, GCNCs and CDPK were observed to be up-regulated in the resistant genotypes (Fig. 6), suggesting that these proteins may play a role in resistance to BW in peanut.

### Further research is required on the WRKY family in relation to bacterial wilt resistance

Transcription factors, especially WRKY transcription factors, are essential for the orderly transmission of signaling pathways in plant immune responses [51–53]. Further study on the function of WRKY transcription factors in the plant immune response will contribute to an improved understanding of the mechanism of plant resistance regulation. Many WRKY transcription factors associated with resistance to bacterial wilt have been identified in diverse plant species, such as Arabidopsis [54], pepper [55], potato (*Solanum tuberosum*) [56] and tomato [57]. However, in the current study, we identified a WRKY gene only expressed in the susceptible genotypes, and a WRKY gene was located within the target QTL region but was not expressed in all genotypes analyzed. Thus, whether a WRKY gene is associated with BW resistance in peanut requires further research.

## Conclusion

In this work, we used full-length transcriptome data for peanut to generate two transcript pools, and RNA-seq analysis was performed on four materials in five periods. The plant-pathogen interaction pathway was identified as a way in which BW-resistant materials prevent invasion by the pathogen. In the BW-susceptible materials, glutathione metabolism may participate in the initial response to pathogen infection. Building on our previous research, the expression levels of the genes located in a major QTL region associated with disease resistance were further analyzed. The genes *arahy.V6I7WA* and *arahy.MXY2PU* may be associated with resistance to BW.

## Methods

### Plant materials and *R. solanacearum* inoculation

A peanut RIL population (F_10_) of 521 lines was developed from a cross between ‘Yuanza 9102’ (female parent) and ‘wt09-0023’ (male parent) by single-seed descent [5]. ‘Yuanza9102’ (Y) is a widely grown cultivar with strong and stable resistance to BW, whereas ‘wt09-0023’ (W) is a susceptible accession. The lines P757 (R, resistant to BW) and P629 (S, susceptible to BW) were selected from the RIL population because they had the most similar genetic background but exhibited opposite resistance to BW. The four materials (Y, W, R, and S) were grown in vermiculite in an artificial climate chamber with a temperature of 30 °C/25°C (day/night), a photoperiod of 16 h/8 h (light/dark), and relative humidity of 60% at the Henan Academy of Agricultural Sciences (Henan, China).

A virulent *R. solanacearum* isolate was obtained from the Institute of Plant Protection, Henan Academy of Agricultural Sciences. The bacteria were streaked on 1% TTC agar medium (containing 10.0 g L^− 1^ peptone, 1 g L^− 1^ casein hydrolysate, 10.0 g L^− 1^ D-glucose, and 18.0 g L^− 1^ agar) and incubated. Monoclones were picked with a sterile tip and incubated in TTC fluid medium in a shaker at 200 rpm at 28 °C for 2 days. At the three- or four-leaf stage, seedlings were inoculated with *R. solanacearum* (OD_600_ = 0.5) by wounding the taproot. Sterile water was used for the mock control.

### Sample collection

A resistance pool (R1) and a susceptible pool (S1) were established for isoform-sequencing (Iso-Seq). Leaves and roots of Y, W, R, and S sampled at 0, 6, 12, 18, 24, 36, 48, 60, 72, 96, 144, and 188 hpi were collected individually for full-length transcriptome analysis. The taproots of the four materials were collected at 0, 12, 24, 48, and 72 hpi; each sample comprised three replicates for RNA-seq analysis. All samples were immediately frozen in liquid nitrogen and stored at − 80 °C.

### RNA extraction, library preparation, and sequencing

Total RNA from each sample was extracted using the TaKaRa MiniBEST Plant RNA Extraction Kit (TaKaRa Bio Inc., Kusatsu, Shiga, Japan). The RNA integrity value (RIN) was checked with a Bioanalyzer 2100 (Agilent Technologies Co., Ltd., Palo Alto, CA, USA). The total RNA quality was assessed by means of 1.2% agarose gel electrophoresis. The RNA samples with RIN > 8 were used for subsequent analyses.

For the Iso-Seq analysis, RNA from the Y and R samples were extracted separately and then mixed equally into one sample (designated R1). Similarly, the W and S extracts were mixed to form the S1 sample. The Iso-Seq library was prepared in accordance with the isoform sequencing protocol using the Clontech SMARTer PCR cDNA Synthesis Kit and the BluePippin Size Selection System protocol, as prescribed by Pacific Biosciences (PN 100-092-800-03), on a PacBio platform by Novogene Co., Ltd. (Beijing, China).

Total RNA from the taproots of the four materials was extracted for each sample and used as the input material to create sequencing libraries using the NEBNext Ultra^TM^ RNA Library Prep Kit for Illumina (NEB, Ipswich, MA, USA) [58], following the manufacturer’s instructions, and deep sequenced by Novogene Co., Ltd. (Beijing, China) using an Illumina sequencing platform.

### PacBio Iso-Seq analysis

The Iso-Seq raw data were processed following the PacBio Iso-Seq pipeline using SMRT Link v8.0 (https://www.pacb.com/support/software-downloads). First, the CCSs were obtained with the CCS algorithm from the subreads BAM file. Next, the CCSs were classified into full-length non-chimeric (FLNC) and non-full-length (nFL) reads based on cDNA primers and the poly-A tail. Subsequently, a consensus sequence was obtained with the hierarchical n*log(n) algorithm by clustering FLNC sequences of the same transcript. Finally, high-quality (HQ) consensus sequences were obtained for further analysis. The Illumina RNA-seq data were used to correct errors in the HQ sequences using LoRDEC-v0.7 (http://atgc.lirmm.fr/lordec). The redundant sequences were removed using CD-HIT-v4.6.8 [59].

After merging all R1 and S1 transcripts, and removal of the redundant sequences, the retained transcripts served as a reference transcriptome. The transcriptome cluster analysis was repeated. The unique and common transcripts among all samples were analyzed based on the cluster analysis results.

### RNA-seq data analysis

The clean reads for each sample obtained from the Illumina sequencing were then compared with the reference transcriptome. In this process, RSEM-v1.3.0 software [60] was used and the parameters for the comparison were set with Bowtie2-v2.3.4 software (http://bowtie-bio.sourceforge.net/bowtie2/index.shtml) in RSEM as an end-to-end and sensitive model. The RSEM software provided statistics on the comparison results. In addition, the read counts for each sample compared with each gene were obtained and converted to FPKM to analyze the gene expression level.

### Functional annotation

The reference transcriptome was compared with the nr [61] and nt databases of the National Center for Biotechnology Information (https://www.ncbi.nlm.nih.gov/), the Swiss-Prot database [62], KOG database [63], and the KEGG database [64] using BLAST software. The Hmmscan software was used for the search of the Pfam database (http://pfam.sanger.ac.uk/). GO [65] terms were assigned using a custom protein annotation script based on the Pfam database annotation.

The BLAST software was used, with the e-value set to ‘1e-10’, for analysis of the nt database. The Diamond software was used for BLASTX searches, with the e-value set to ‘1e-10’, of the nr, KOG, Swiss-Prot, and KEGG databases.

### Gene structure analysis

The CDS and protein sequences were predicted using the ANGEL-v2.4 software [66]. Transcription factors are a group of protein molecules that show specifically binding to sequences upstream of the 5’ end of genes, thus ensuring the expression of target genes with specific intensity at a specific time and space. We used iTAK software [67] to predict plant transcription factors.

### Differential gene expression analyses and enrichment analysis

The DEGs were identified using DESeq2 [68], using thresholds of false discovery rate < 0.05 and |log_2_ (fold change)| > 1. The GOseq-v1.10.0 software (http://www.bioconductor.org/packages/release/bioc/html/goseq.html) was used for GO enrichment analysis of the DEGs. KOBAS-v3.0 software (http://kobas.cbi.pku.edu.cn/download.php) was used for KEGG pathway enrichment analysis.

### Candidate gene mapping to the unpublished genome

In total, 600 K sequences, including the candidate region of the Tifrunner genome and the unpublished Yuanza 9102 genome, were extracted separately as a new reference. The S1 transcripts were aligned to the extracted Tifrunner sequence, whereas R1 transcripts were aligned to the extracted new Yuanza 9102 genome. The name designated to a gene was determined by the position of the transcript on the genome.

The length of 180 and 273 K sequences of the unpublished Yuanza 9102 genome and the Tifrunner genome were extracted separately for assessment of collinearity. Collinearity was analyzed using the MUMmer software.

### Quantitative real-time PCR validation of differentially expressed genes

To validate the RNA-seq results, qRT-PCR analyses were performed. Several candidate transcriptomes were selected for qRT-PCR analysis using three biological replicates. Gene-specific primers were designed using Primer 5.0 and were commercially synthesized (Qinco, Beijing, China). First, RNA was reverse-transcribed to cDNA using the Reverse Transcription System A3500 (Promega, Madison, WI, USA). Next, the qRT-PCR was performed in triplicate for each sample using the PowerUp SYBR Green Master 2× Mix (Thermo Fisher Scientific, Waltham, MA, USA) following the manufacturer’s instructions. Each qRT-PCR amplification was performed using the StepOne Real-Time PCR System and the associated StepOne software (Thermo Fisher Scientific).

### Electronic supplementary material

Below is the link to the electronic supplementary material.

Supplementary Material 1

Supplementary Material 2

## Data Availability

The sequence data is deposited in NGDC database under GSA accession: PRJCA018886.
