# Supplementary material for: Transcriptome sequencing and expression analysis in peanut reveal the potential mechanism response to Ralstonia solanacearum infection
Source: BMC Plant Biol. 2024 Mar 21;24:207. doi: 10.1186/s12870-024-04877-0 (PMC10956345; doi:10.1186/s12870-024-04877-0)
Supplement: Supplementary file 1 — Supplementary Material 1 [file 12870_2024_4877_MOESM1_ESM.docx]

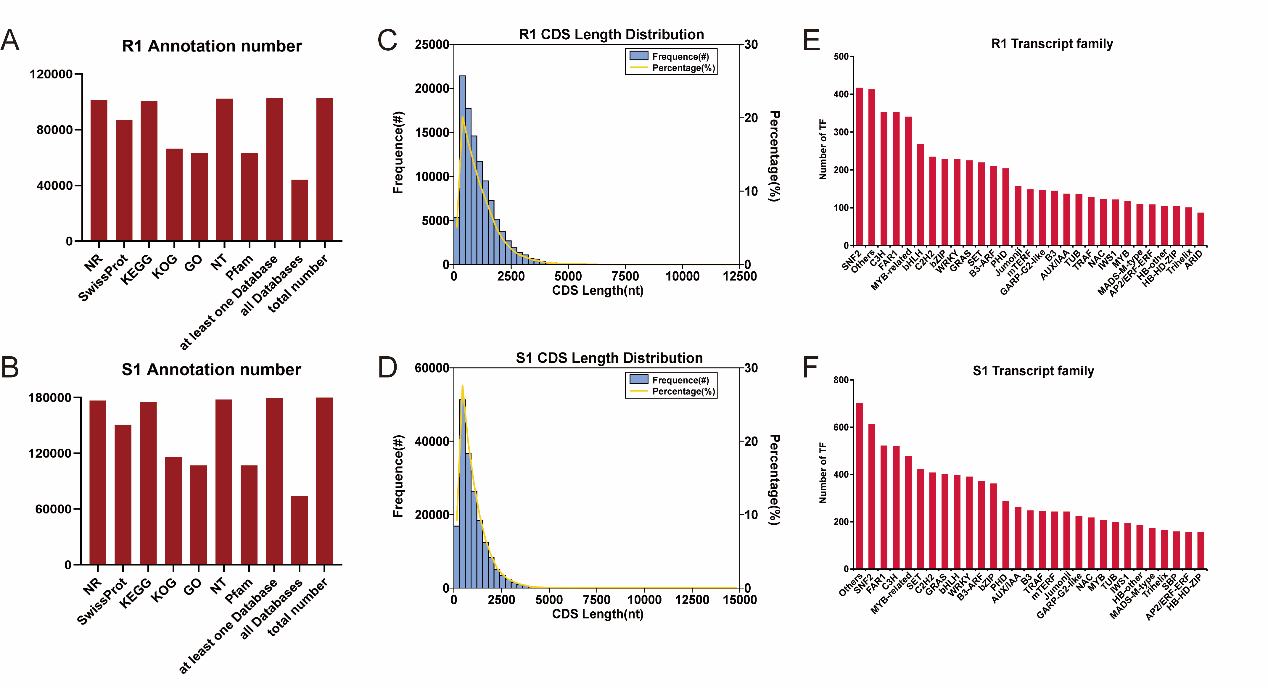


Fig. S1 Full-length transcriptome analysis. Annotation of (A) R1 and (B) S1 transcripts with information from eight public databases. Coding sequence (CDS) length distribution for (C) R1 and (D) S1 transcripts. Number of transcription factor families identified among (E) R1 and (F) S1 transcripts.


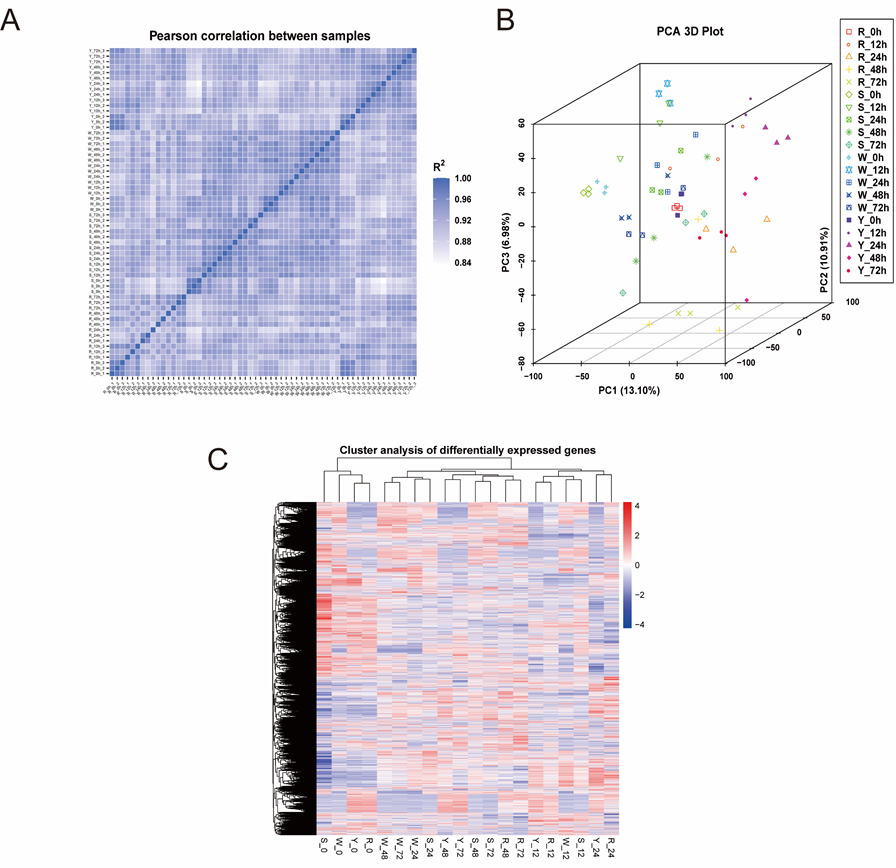


Fig. S2 Sample correlation analysis. (A) Pearson correlation analysis between all samples. (B) Principal component analysis (PCA) of all samples of peanut materials in five periods after inoculation with Ralstonia solanacearum. (C) Cluster analysis of differentially expressed genes.


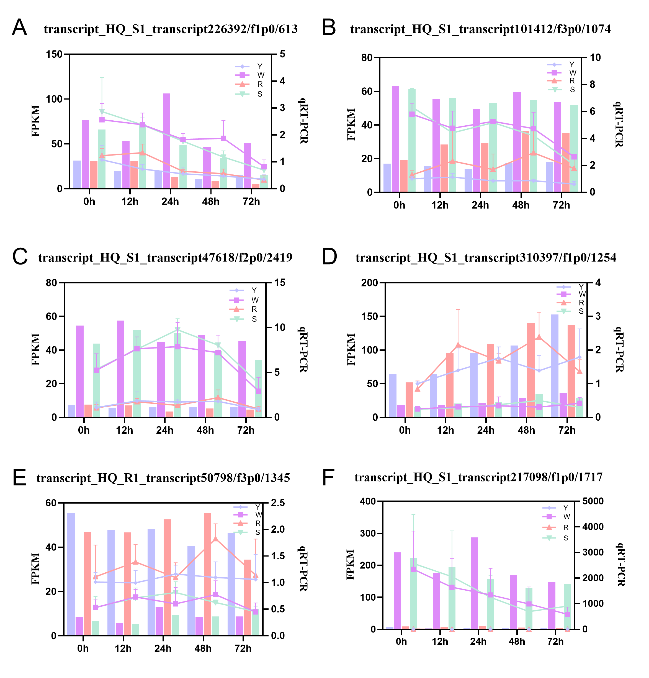


Fig. S3 Quantitative real-time reverse transcriptase PCR (qRT-PCR) validation of six differentially expressed genes (DEGs) associated with resistance to bacterial wilt. (A-F) The horizontal coordinate is the developmental period and the vertical coordinate is the relative gene expression level. The lines indicate the relative expression determined by qRT-PCR and the columns indicate the expression level (FPKM) determined by RNA-sequencing. The relative expression levels were estimated from the threshold of the PCR cycle using the 2^−△△Ct^ method. Error bars indicate the standard errors from three independent biological and three technical replicates for the qRT-PCR data.

| **Table S1 Statistics of polymerase read** | | | | |
| --- | --- | --- | --- | --- |
| **Sample** | **Polymerase Read Bases(G)** | **Polymerase Reads** | **Polymerase Read Length (mean)** | **Polymerase Read N50** |
| R1 | 73.49 | 644156 | 114088 | 189634 |
| S1 | 165.09 | 1547052 | 106716 | 182792 |

| **Table S2 Statistics of Subreads** | | | | |
| --- | --- | --- | --- | --- |
| **Sample** | **Subreads base(G)** | **Subreads number** | **Average subreads length** | **N50** |
| R1 | 71.37 | 29093036 | 2454 | 2687 |
| S1 | 159.91 | 68500115 | 2335 | 2549 |

| **Table S3 Statistics of classify** | | | | | | | | |
| --- | --- | --- | --- | --- | --- | --- | --- | --- |
| **Sample** | **CCS number** | **mean number passes of CCS** | **NFL** | **FL** | **FLNC** | **FLC** | **mean readlength of FLNC** | **FLNC/CCS** |
| R1 | 596303 | 9 | 62878 | 533425 | 528760 | 4665 | 2583 | 0.8867 |
| S1 | 1411238 | 9 | 155271 | 1255967 | 1250648 | 5319 | 2412 | 0.8862 |
|  |  |  |  |  |  |  |  |  |

| **Table S4 Statistics of Polished Consensus** | | | | | |
| --- | --- | --- | --- | --- | --- |
| **Sample** | **Consensus number** | **Min length** | **Max length** | **Mean length** | **N50** |
| R1 | 53927 | 59 | 8817 | 2576 | 2789 |
| S1 | 104003 | 59 | 8563 | 2420 | 2635 |
|  |  |  |  |  |  |

| **Table S5 Summary of samples sequencing data quality** | | | | | | | | | |
| --- | --- | --- | --- | --- | --- | --- | --- | --- | --- |
| **Sample** | raw_reads | raw_bases | clean_reads | clean_bases | error_rate | Q20 | Q30 | GC_pct | Mapping rate |
| Y_0h_1 | 45879398 | 6.88G | 44633540 | 6.7G | 0.03 | 97.76 | 93.64 | 44.68 | 37775640(84.64%) |
| Y_0h_2 | 46832686 | 7.02G | 45077062 | 6.76G | 0.03 | 97.93 | 93.97 | 44.13 | 38123150(84.57%) |
| Y_0h_3 | 44856210 | 6.73G | 43127892 | 6.47G | 0.03 | 97.92 | 93.96 | 43.96 | 36391342(84.38%) |
| W_0h_1 | 45597644 | 6.84G | 44372368 | 6.66G | 0.03 | 97.76 | 93.59 | 44.11 | 37182724(83.80%) |
| W_0h_2 | 43313254 | 6.5G | 41824150 | 6.27G | 0.03 | 97.81 | 93.7 | 44.16 | 35113268(83.95%) |
| W_0h_3 | 44201366 | 6.63G | 42692590 | 6.4G | 0.03 | 97.76 | 93.56 | 44.08 | 35561050(83.30%) |
| S_0h_1 | 41747354 | 6.26G | 40247846 | 6.04G | 0.03 | 97.95 | 94 | 44.01 | 33680488(83.68%) |
| S_0h_2 | 41553302 | 6.23G | 40385816 | 6.06G | 0.03 | 97.84 | 93.72 | 43.95 | 34110202(84.46%) |
| S_0h_3 | 43766352 | 6.56G | 42714542 | 6.41G | 0.03 | 97.79 | 93.6 | 43.71 | 36401718(85.22%) |
| R_0h_1 | 44170806 | 6.63G | 42986560 | 6.45G | 0.03 | 97.7 | 93.46 | 44.29 | 35911688(83.54%) |
| R_0h_2 | 43809008 | 6.57G | 42418660 | 6.36G | 0.03 | 97.95 | 94.04 | 44.16 | 36091784(85.08%) |
| R_0h_3 | 44378154 | 6.66G | 43218500 | 6.48G | 0.03 | 97.8 | 93.69 | 44.53 | 36392198(84.21%) |
| Y_12h_1 | 45971924 | 6.9G | 44692190 | 6.7G | 0.03 | 97.56 | 93.35 | 45.54 | 38467672(86.07%) |
| Y_12h_2 | 43556932 | 6.53G | 41813032 | 6.27G | 0.03 | 97.85 | 93.84 | 44.97 | 35251782(84.31%) |
| Y_12h_3 | 43673624 | 6.55G | 42478686 | 6.37G | 0.03 | 97.88 | 93.85 | 44.31 | 36198614(85.22%) |
| W_12h_1 | 45873982 | 6.88G | 45334198 | 6.8G | 0.03 | 97.8 | 93.76 | 44.62 | 38491418(84.91%) |
| W_12h_2 | 46286532 | 6.94G | 45122292 | 6.77G | 0.03 | 97.82 | 93.77 | 44.73 | 37979734(84.17%) |
| W_12h_3 | 45111208 | 6.77G | 43783194 | 6.57G | 0.03 | 97.82 | 93.76 | 44.44 | 37173234(84.90%) |
| S_12h_1 | 43644632 | 6.55G | 41979328 | 6.3G | 0.03 | 97.78 | 93.76 | 44.51 | 35557472(84.70%) |
| S_12h_2 | 44039304 | 6.61G | 42792476 | 6.42G | 0.03 | 97.9 | 93.94 | 44.24 | 36272762(84.76%) |
| S_12h_3 | 47066856 | 7.06G | 46059568 | 6.91G | 0.03 | 97.67 | 93.4 | 44.42 | 38950528(84.57%) |
| R_12h_1 | 45329624 | 6.8G | 44244490 | 6.64G | 0.03 | 97.71 | 93.48 | 45.07 | 37622500(85.03%) |
| R_12h_2 | 44795954 | 6.72G | 43633350 | 6.55G | 0.02 | 98.09 | 94.38 | 44.15 | 36783742(84.30%) |
| R_12h_3 | 44521064 | 6.68G | 43478282 | 6.52G | 0.03 | 97.61 | 93.27 | 44.78 | 36767866(84.57%) |
| Y_24h_1 | 48131042 | 7.22G | 46812272 | 7.02G | 0.03 | 97.53 | 93.13 | 44.58 | 39489366(84.36%) |
| Y_24h_2 | 45405004 | 6.81G | 44639008 | 6.7G | 0.03 | 97.91 | 93.92 | 44.39 | 38234870(85.65%) |
| Y_24h_3 | 41213814 | 6.18G | 40521786 | 6.08G | 0.03 | 97.71 | 93.43 | 44.24 | 34767868(85.80%) |
| W_24h_1 | 46795588 | 7.02G | 46083868 | 6.91G | 0.03 | 97.83 | 93.68 | 44.35 | 39783638(86.33%) |
| W_24h_ | 44246486 | 6.64G | 42282124 | 6.34G | 0.03 | 97.69 | 93.39 | 44.15 | 35271686(83.42%) |
| W_24h_3 | 44149650 | 6.62G | 43216462 | 6.48G | 0.03 | 97.96 | 93.97 | 43.98 | 36922914(85.44%) |
| S_24h_1 | 45098920 | 6.76G | 43495298 | 6.52G | 0.03 | 97.79 | 93.59 | 44.14 | 36074924(82.94%) |
| S_24h_2 | 43944512 | 6.59G | 43005534 | 6.45G | 0.02 | 98.07 | 94.26 | 44.34 | 35571158(82.71%) |
| S_24h_3 | 43894832 | 6.58G | 42387774 | 6.36G | 0.03 | 97.9 | 93.82 | 43.9 | 35677584(84.17%) |
| R_24h_1 | 44149498 | 6.62G | 42828848 | 6.42G | 0.03 | 97.91 | 93.86 | 43.03 | 35864698(83.74%) |
| R_24h_2 | 44232620 | 6.63G | 43483768 | 6.52G | 0.03 | 97.61 | 93.19 | 42.93 | 35520302(81.69%) |
| R_24h_3 | 45559800 | 6.83G | 44383106 | 6.66G | 0.03 | 98.05 | 94.1 | 42.69 | 37634692(84.80%) |
| Y_48h_1 | 43895376 | 6.58G | 42744748 | 6.41G | 0.03 | 97.76 | 93.49 | 43.23 | 37209958(87.05%) |
| Y_48h_2 | 45572004 | 6.84G | 44483182 | 6.67G | 0.03 | 97.69 | 93.41 | 44.36 | 37584620(84.49%) |
| Y_48h_3 | 45602436 | 6.84G | 44095264 | 6.61G | 0.03 | 97.82 | 93.68 | 44.23 | 37405460(84.83%) |
| W_48h_1 | 45706412 | 6.86G | 44693404 | 6.7G | 0.03 | 97.74 | 93.46 | 44.02 | 37714186(84.38%) |
| W_48h_2 | 45019162 | 6.75G | 43866012 | 6.58G | 0.03 | 97.63 | 93.23 | 44.21 | 36966920(84.27%) |
| W_48h_3 | 43254700 | 6.49G | 42383922 | 6.36G | 0.03 | 97.58 | 93.11 | 43.94 | 35841440(84.56%) |
| S_48h_1 | 44078284 | 6.61G | 43374050 | 6.51G | 0.03 | 97.71 | 93.41 | 44.01 | 36864922(84.99%) |
| S_48h_2 | 44004298 | 6.6G | 43062828 | 6.46G | 0.03 | 97.64 | 93.21 | 43.45 | 36678264(85.17%) |
| S_48h_3 | 43539940 | 6.53G | 42585196 | 6.39G | 0.03 | 97.71 | 93.36 | 43.09 | 36192548(84.99%) |
| R_48h_1 | 45168592 | 6.78G | 44449836 | 6.67G | 0.03 | 97.88 | 93.72 | 42.94 | 38298964(86.16%) |
| R_48h_2 | 41761894 | 6.26G | 41174406 | 6.18G | 0.03 | 97.47 | 92.79 | 42.82 | 35518236(86.26%) |
| R_48h_3 | 45141918 | 6.77G | 44568974 | 6.69G | 0.03 | 97.97 | 94.12 | 44.05 | 38740814(86.92%) |
| Y_72h_1 | 43762768 | 6.56G | 42822972 | 6.42G | 0.03 | 97.57 | 93.13 | 44.09 | 36409582(85.02%) |
| Y_72h_2 | 44713034 | 6.71G | 43946716 | 6.59G | 0.03 | 97.37 | 92.65 | 43.97 | 37354358(85.00%) |
| Y_72h_3 | 45671076 | 6.85G | 44776182 | 6.72G | 0.03 | 97.66 | 93.3 | 44 | 38149476(85.20%) |
| W_72h_1 | 44357678 | 6.65G | 43198918 | 6.48G | 0.03 | 97.85 | 93.73 | 43.9 | 36357696(84.16%) |
| W_72h_2 | 45094680 | 6.76G | 43724752 | 6.56G | 0.03 | 97.52 | 92.97 | 43.99 | 37282604(85.27%) |
| W_72h_3 | 42250628 | 6.34G | 41504592 | 6.23G | 0.03 | 97.69 | 93.32 | 43.58 | 35414828(85.33%) |
| S_72h_1 | 45133226 | 6.77G | 44544552 | 6.68G | 0.03 | 97.7 | 93.3 | 43.24 | 37909414(85.10%) |
| S_72h_2 | 41254234 | 6.19G | 40745526 | 6.11G | 0.03 | 97.72 | 93.34 | 43.63 | 35358652(86.78%) |
| S_72h_3 | 44454300 | 6.67G | 44019840 | 6.6G | 0.03 | 97.89 | 93.75 | 43.01 | 38386230(87.20%) |
| R_72h_1 | 44596842 | 6.69G | 44089670 | 6.61G | 0.03 | 97.78 | 93.58 | 43.44 | 38822658(88.05%) |
| R_72h_2 | 46444990 | 6.97G | 45805112 | 6.87G | 0.03 | 97.88 | 93.78 | 43.26 | 40199822(87.76%) |
| R_72h_3 | 42124606 | 6.32G | 41323082 | 6.2G | 0.03 | 97.67 | 93.3 | 43.5 | 36275522(87.79%) |
|  |  |  |  |  |  |  |  |  |  |

| **Table S6. qRT-PCR primers for validation of RNA-Seq data**. | | |
| --- | --- | --- |
| **Transcript_ID** | **Primer-Forward** | **Primer-Reverse** |
| transcript_HQ_S1_transcript226392/f1p0/613 | CGCTGGTGTTTCCGATTCTT | CTCTGTTGTGCTGCTGCTCT |
| transcript_HQ_S1_transcript101412/f3p0/1074 | AATGCTATTCAACCTTCGGTAC | GTCTGCCATCTCCAACTCAC |
| transcript_HQ_S1_transcript47618/f2p0/2419 | AAGACCGGAAAGGCCAACG | ACCAGCAATGCCCGCAAC |
| transcript_HQ_S1_transcript310397/f1p0/1254 | TTTGATTTGCCTCGTGTTGT | TATGACCTTCCCATGTGGTG |
| transcript_HQ_S1_transcript217098/f1p0/1717 | AGAGTCATTGGTGATGGGAGTT | TCTGCTTCTGCTGTGCGTGT |
| transcript_HQ_R1_transcript50798/f3p0/1345 | GAACAGAAGAGGAGGAGGCG | TCACCATTGCACTGGGATAA |
| ADH3 | GACGCTTGGCGAGATCAACA | AACCGGACAACCACCACATG |
|  |  |  |
